# Current Management and Future Perspective in Pleural Mesothelioma

**DOI:** 10.3390/cancers14041044

**Published:** 2022-02-18

**Authors:** Rajiv Shah, Laura V. Klotz, Julia Glade

**Affiliations:** 1 Department of Thoracic Oncology, Thoraxklinik, Heidelberg University Hospital, 69126 Heidelberg, Germany; 2 Department of Thoracic Surgery, Thoraxklinik, Heidelberg University Hospital, 69126 Heidelberg, Germany; laura.klotz@med.uni-heidelberg.de; 3 Institute for Pathology, Heidelberg University Hospital, 69120 Heidelberg, Germany; julia.glade@med.uni-heidelberg.de

**Keywords:** mesothelioma, immunotherapy, surgery, biomarker, chemotherapy, checkpoint, multimodal treatment

## Abstract

**Simple Summary:**

Platin-based chemotherapy with pemetrexed has been the backbone of meaningful treatment in pleural mesothelioma for the last 16 years, alongside vinorelbine and gemcitabine, which show only modest response. Recently, a nivolumab and ipilimumab combination was approved, and it offers improvement in survival rate and quality of life. With the identification and understanding of novel biomarkers, many treatment options are currently being evaluated.

**Abstract:**

Pleural mesothelioma is an aggressive malignancy arising from pleural mesothelial cell lining, predominantly associated with prior exposure to asbestos. The ban on asbestos use has led to its lower incidence in many countries, but globally the disease burden is expected to rise. Therefore, well-planned research is needed to develop more effective, tolerable and affordable drugs. The development of novel treatment has been too slow, with only two regimens of systemic therapy with robust phase 3 data approved formally to date. The treatment scenario for resectable disease remains controversial. However, recent developments in the understanding of disease and clinical trials have been encouraging, and may add better treatment options in the coming years. In this review, we discuss the current treatment options for pleural mesothelioma and shed light on some recent studies and ongoing trials.

## 1. Introduction

Malignant pleural mesothelioma (MPM) arises due to chronic inflammation and malignant cellular proliferation of the mesothelial cell lining of the pleura. In 70–80% of the cases, there is a known history of exposure to asbestos, a natural mineral historically used for multiple industrial and household purposes [1]. The long period of latency causes the disease onset 20–50 years after exposure. Even though the mining and industrial use of asbestos has been banned or strictly regulated in many countries, including those in the European Union, Russia, Kazakhstan and China contribute to more than 80% of the global production. Global consumption remains high particularly in swing states, where the import and usage of asbestos without adequate safety equipment will inevitably lead to a rise in global disease burden in the coming decades [2,3,4]. Well-conducted clinical trials, investment in research, and development of new and more effective drugs that are affordable globally are the current requirements to improve the treatment landscape in mesothelioma.

Until the landmark trial by Vogelzang et al. in 2003, which showed that a combination of cisplatin and pemetrexed improves the overall survival (OS) and quality of life (QoL), the treatment landscape of mesothelioma was dominated by best supportive care (BSC), single-agent chemotherapy, in select cases radical surgery, and/or radiotherapy (RT) [5,6,7,8,9,10]. Another decade later, a MAPS study showed further improvement in OS with the addition of bevacizumab, an anti-VEGF-inhibitor [11]. With the advent of immune checkpoint inhibitors (ICI) in solid tumors, multiple studies in mesothelioma showed some promising results with anti-programmed cell death protein 1 (anti-PD1) or anti-programmed death-ligand 1 (anti-PD-L1) inhibitors as a single agent or in combination with anti-cytotoxic T-lymphocyte-associated protein 4 (anti-CTLA4) inhibitor [12,13,14,15,16,17]. However, the latter as a single agent did not show any benefit when compared with placebo [18].

Since 2020, the combination of nivolumab and ipilimumab has represented the most effective treatment in pleural mesothelioma [19]. The benefit is even more robust in tumors of the non-epithelioid subtype, which is generally attributed as a disease with high tumor burden, rapid progression, chemotherapy resistant and the worst prognosis, to mention a few. Interestingly, the combination of ICI, in contrary to chemotherapy, does not seem to discriminate much between the subtypes in pleural mesothelioma, leading to much better treatment options for patients with non-epithelioid histology [20,21]. Despite the low tumor mutational burden in pleural mesothelioma, recent studies suggest immunologic mechanisms in the suppression of tumor growth. This provides a basis for further investigation of combination regimens of ICI with other drugs (e.g., vascular endothelial growth factor (VEGF) inhibitor, focal adhesion kinase (FAK) inhibitor, poly (ADP-ribose) polymerase (PARP) inhibitors, etc.). Furthermore, modern cell-based treatment strategies are under investigation, which include but are not limited to chimeric antigen receptor (CAR)-T cell therapy, dendritic cell therapy and oncolytic viral therapy [22,23,24,25,26,27].

Whether there is any role for multimodal treatment is a matter of current research [28,29,30]. Such an approach is recommended by major international guidelines and should be conducted in dedicated high volume mesothelioma centers with specific multidisciplinary expertise, including surgery. To make such individual treatment approach accessible to all patients, so that selected patients can receive multimodal treatment, special oncological structures with regional network and cooperation are needed. In 2021, the German Cancer Society started accrediting mesothelioma unit certification along with the certification of lung cancer centers. Such programs not only help generate awareness on expert centers and offer the best possible treatment within clinical trials for such rare diseases but also mitigate the risk of undertreatment, especially when the disease is resectable. They also help with palliative care and advanced care planning. 

The role of radiotherapy (RT) in a multimodal setting is currently under investigation. While RT is recommended for analgesic symptom control, prophylactic irradiation of the procedure track is not routinely recommended [31].

While new treatment strategies are investigated, these approaches must be accompanied by modern molecular pathologic methods to identify predictive and prognostic biomarkers and tailor the best suited medicine.

## 2. Histological and Molecular Characteristics

### 2.1. Histomorphology, Histological Assessment and Molecular Classification

MPM are divided into three histological subtypes: epithelioid, biphasic and sarcomatoid [32]. The biphasic subtype consists of varying amounts of epithelioid and sarcomatoid components [33,34]. Histomorphological grading for epithelioid MPM is a two-tiered system (“low grade” and “high grade”) based on recent publications by the EURACAN/IASLC consensus, nuclear pleomorphism, and extent of necrosis and mitotic activity [35]. Currently, no viable grading system for sarcomatoid or biphasic MPM exists. The mechanism driving differentiation into either histological subtype is largely unknown. Quantification of sarcomatoid MPM components is recommended, although transitional tumor areas exist and are subject to notable interobserver variability [36]. Alcala et al. propose to redefine MPM types as a continuum driven by the interaction between immune response and vascular factors, rather than adhering to distinct subgroups [37]. Some studies even suggest reversibility between epithelioid and sarcomatoid MPM components [38].

Concerning cytological and stromal patterns of architecture, efforts have been made to classify and quantify these as is standard for lung adenocarcinoma [39,40], since some architectural patterns seem to influence prognosis and can potentially lead to misdiagnosis as they mimic carcinoma [35]. In epithelioid MPM, microcystic or tubulopapillary growth patterns are associated with the longest overall survival when compared to trabecular or solid variants [41,42,43].

Histological assessment of MPM includes a standard immunohistochemical panel consisting of at least two mesothelial and two carcinoma markers in accordance with the most recent WHO classification of thoracic tumors (2021), the main differential diagnosis being metastatic carcinoma [32]. Calretinin, D2-40 (podoplanin), Wilms’ tumor-1 (WT1) and CK5/6 are recommended for distinguishing mesothelial differentiation, whereas the common markers of carcinoma are BerEP4, CEA (carcinoembryonic antigen), pancytokeratins and, more recently, claudin-4 as a marker of adenocarcinoma. Squamous markers such as p40 and p63 distinguish squamous cell carcinoma from mesothelioma with squamoid features; however, exceptionally rare cases of MPM appear to show true squamous differentiation as well. Immunohistochemical markers specific to the anatomic site of origin can be useful in identifying metastatic carcinoma of the gastrointestinal tract, lung, breast, kidney or prostate, as well as melanoma. GATA3 immunohistochemistry can be a pitfall since some MPM cases have shown positivity [44,45]. 

Differential diagnosis further includes localized, well-differentiated papillary mesothelioma (WDPM), a potentially resectable disease with a less severe prognosis than MPM [46]. WDPM cannot be distinguished from MPM on histology alone. Instead, assessment of genetic alterations common to MPM is required for classification. Furthermore, WDPM cannot be diagnosed if invasive growth is reported according to the current WHO classification of thoracic tumors [32].

De Reynies et al. first attempted to classify MPM by using transcriptome data, resulting in two molecular subgroups of MPM cell cultures [47]. Their efforts resulted in two robust subgroups, named C1 and C2, which have distinct characteristics with regard to molecular profiles, gene alterations as well as clinical outcomes. Two years later, Bueno et al. described four molecular subgroups based on gene expression profiling [33], similar to Hmeljak et al., who analyzed mRNA expression in MPM [48]. Bueno et al. managed to separate four clusters of MPM—epithelioid, sarcomatoid, biphasic-epithelioid and biphasic-sarcomatoid—with non-biphasic clusters emerging as the most distinctly different ones. These clusters closely resemble the histomorphological MPM subtypes. Therefore, multi-platform analysis efforts have been made by Hmeljak et al. to add prognostic value to molecular profiling in MPM, resulting in several prognostic subgroups as defined by PARADIGM and iCluster. These subgroups range between good and poorer prognosis, while varying between genetic alterations, miRNA expression and immune cell signature [48].

Preliminary data from smaller cohorts demonstrated an overexpression of PD-L1 in sarcomatoid MPM [33]. In contrast, epithelioid MPM tends to overexpress VISTA (V-domain Ig suppressor of T cell activation), an immune checkpoint gene that inhibits anti-tumor immune response [48]. 

### 2.2. Molecular Aberrations

Little is known about the spectrum and prevalence of genetic alterations, particularly macroaberrations, in MPM. The main reason for this is the rarity of the disease, resulting in a very limited sample number for genomic studies. In recent years, two studies with molecular analyses in larger MPM cohorts have been published. Firstly, Bueno et al. analyzed 216 cases. Secondly, scientists of the Cancer Genome Atlas project (TCGA) conducted an integrative genomic analysis. Based on their results, the most common genetic aberrations according to current knowledge are deleterious mutations in *BAP1* (*BRCA1* associated protein 1), *CDKN2A* and *NF2* [33,48,49], as well as copy number variations [48]. *BAP1* is a deubiquitinating enzyme that is encoded by the *BAP1* gene, a tumor suppressor gene, in humans, playing a role during DNA repair [50,51]. In MPM, it has a mutation rate of 38% according to the TCGA. However, mutation rate of up to 60% has been reported by other authors [51,52]. Its influence on the development of MPM is not sufficiently understood and may be more complex than expected. Correlation between immunohistochemistry and molecular aberrations of *BAP1* as well as distinct expression profiles in histological subtypes is of significant interest, although no such association has been successfully proved yet. De Rienzo et al. provided a large-scale analysis of *BAP1* in 596 MPM tumors, demonstrating nuclear positivity and wild-type expression of *BAP1* predominantly in sarcomatoid MPM, a significantly lower mutation rate compared to epithelioid and biphasic subtypes consistent with these findings [52]. Up to 77% of the epithelioid subtype show an alteration of *BAP1*, compared to only up to 15% in sarcomatoid MPM [53,54]. In addition, sarcomatoid tumor cells in biphasic MPM can retain nuclear expression of *BAP1* [55]. However, atypical stroma cells in biphasic MPM can exhibit nuclear expression of *BAP1* as well while epithelioid tumor cells stain negatively, posing a diagnostic challenge to distinguish atypical stromal cells from neoplastic cells and categorize the tumor accordingly [56,57].

*CDKN2A*, a gene known as cyclin-dependent kinase inhibitor 2A, codes for two proteins whose main function is tumor suppression, one of which is p16, whose role in tumorigenesis is well established [58,59]. Homozygous deletion of p16/*CDKN2A* is very common in MPM, and is associated with worse prognosis [60,61,62]. Between 30% and 74% of MPM patients show alterations in *CDKN2A* [63,64,65].

Genetic risk factors seem to interact with asbestos exposure in such a way that lower cumulative exposure is needed to develop MPM when a germline mutation in *BAP1* is present [66]. Betti et al. found a similar type of interaction for *CDKN2A*, specifically concerning the germline mutation p.Gly101Trp [67]. Germline mutations in *CDKN2A* as well as *BAP1* carry not only an increased risk for the development of MPM, but also for uveal or cutaneous melanoma and, in the case of *BAP1*, a wide range of other tumors. The BAP1 tumor predisposition syndrome (BAP1-TPDS) has been characterized and associated with an increased risk for BAP1-inactivated melanocytic tumors (formerly known as atypical Spitz tumors), renal cell carcinoma, basal cell carcinoma, hepatocellular carcinoma, cholangiocarcinoma, meningioma as well as a number of other tumors suspected but not yet confirmed to be related to BAP1-TPDS [68]. Carbone et al. currently recommend both germline and tumor cell *BAP1* testing in patients with mesothelioma, renal cell carcinoma and uveal melanoma to aid diagnosis and clinical treatment. Once targeted therapies geared towards *BAP1* become available for these patients, *BAP1* mutational status—both somatic and germline—may become an important tool in assessing therapeutic options and individual prognosis [69]. 

In recent studies, *NF2* and *LATS2*, both belonging to the hippo pathway, were identified as key drivers for non-asbestos-related tumorigenesis for mesothelioma [70], although both also play a role in patients with mesothelioma who were exposed to asbestos. Mouse models showed asbestos-independent development of mesothelioma in populations with *NF2* mutations [71,72]. Alterations in *NF2* can be found in 19–53% of patients with MPM, while mutations in *LATS2* have been reported in 11% of MPM [33,64,73].

### 2.3. Tumor Microenvironment and Heterogeneity

Most current studies emphasize an insufficient extent of knowledge regarding the tumor microenvironment (TME) in MPM, especially the role that varying immune cell populations play in the process. Preliminary data indicate distinctive variability. Independent from histological subtypes, intertumoral heterogeneity is pronounced, particularly regarding B cells, M2 macrophages and CD8+ T cells [37]. An immunosuppressive environment of reduced CD4- and CD8+ T cells promotes tumor invasion, tumor growth and tumor escape [74], thereby aiding the development of immune checkpoint inhibitor resistance [75]. On the other hand, high levels of tumor-infiltrating lymphocytes (TIL) are associated with improved survival [76]. Recently, Zhang et al. demonstrated how clonal architecture and evolutionary clusters influence immune landscape and immune escape mechanisms, providing an additional prognostic factor to immune checkpoint inhibition response while also proving a prognostic impact for certain evolutionary clusters that are associated with poorer prognosis [65].

MPM is prone to high tumor heterogeneity, making it difficult to develop effective targeted therapies but also opening up new possibilities where standard chemotherapy options have been exhausted [77]. Efforts are being made to understand the role of the tumor microenvironment (TME) on tumor proliferation, drug resistance and its interplay with immune cells [78,79,80]. Particularly biphasic subtypes show notable intertumoral and intratumoral heterogeneity, with sarcomatoid and epithelioid components varying between 10–90%. Therefore, multiple biopsies at the time of initial diagnosis can provide an advantage in documenting tumor components sufficiently [81]. Heterogeneity extends to the molecular level as well. While some patients astonishingly keep the same *BAP1* mutation over a time span of 13 years [70], in other cases, heterogeneous variants of *NF2* mutations are present even in two samples of a given patient taken at the same time [82]. Platin-based chemotherapy can influence tumor heterogeneity [83] in various ways, such as a reduction in various proteins belonging to the PI3K-mTOR pathway or increased expression of *NF2* in yet-unpublished data of the same group.

## 3. Surgical Diagnostic and Treatment

### 3.1. Videothoracoscopy as Gold Standard

Cytologic analysis of pleural fluid after diagnostic or therapeutic pleural puncture is often inconclusive, with correct diagnosis in only 25%. In particular, a reliable differentiation between the individual subtypes is rarely possible [84]. However, this subtype differentiation is crucial for further therapy planning. Therefore, according to the guidelines of the European Respiratory Society and the European Society of Thoracic Surgeons, video-assisted thoracoscopy (VATS) with thoracoscopic exploration and multiple biopsies of sufficiently representative tumor tissue remains the gold standard for histopathological diagnosis [85]. During routine uniportal VATS, visual assessment of tumor extension on visceral and parietal pleura as well as the diaphragm and pericardium can be performed in addition to representative tissue sampling to evaluate possible surgical treatment options in a multimodal treatment approach. Therefore, thoracoscopy should be performed at an experienced center [86,87].

### 3.2. Surgical Treatment in Multimodal Setting

The role of radical surgery in a multimodal treatment strategy remains controversial since a satisfying confirmatory result from a phase 3 study is still lacking, and prior attempts to answer the role of surgery in mesothelioma have not been without controversy [28,29,30,88]. However, prior studies have also reported improved survival after extended pleurectomy and decortication (ePD) [89,90,91] as the more radical and morbid technique, extrapleural pneumonectomy (EPP), is being abandoned increasingly [92,93,94]. Moreover, various non-standardized surgical techniques make a comparison even more challenging [95]. While a phase 3 randomized trial (MARS-2, ClinicalTrials.gov identifier, NCT02040272) evaluating ePD to no surgery is still ongoing, selected patients should be encouraged to participate in a clinical trial whenever possible. An individual treatment modality should otherwise only be offered in a high-volume center with dedicated multi-disciplinary mesothelioma experts [31,85,96,97].

In EPP, a so-called en bloc resection of the lung with visceral and parietal pleura is performed. In the case of tumor involvement in the pericardium and diaphragm, the structures are also resected and, depending on the size of the resulting defect, replaced with a membrane. Various studies show a satisfactory survival rate for patients after completion of the trimodal therapy concept of chemotherapy, surgery and radiotherapy. A high perioperative morbidity of 22% to 82% is described with mortality rates at 2% to 6.8% in experienced centers [98]. However, this procedure is only suitable for patients with excellent pulmonary function and a lack of prohibiting comorbidities. Due to the long latency period from asbestos exposure to disease manifestation, an increasingly older patient population with various comorbidities, especially cardiac comorbidities, has become apparent in recent years. In order to be able to offer these patients a surgical therapy approach in the multimodal therapy concept despite the presence of comorbidities, a lung parenchyma-sparing surgical procedure has become the surgical procedure of choice worldwide in recent years [31]. During pleurectomy and decortication (PD), the entire parietal and visceral pleura is resected and the lung parenchyma spared. In the case of tumor involvement, the so-called ePD also involves diaphragmatic and/or pericardial resection. Depending on the size of the resection defect created to achieve the macroscopic complete resection (MCR), replacement of the structures is performed. Due to the preservation of the lung parenchyma, good vital capacity and improved one-second forced expiratory volume (FEV1) can be achieved postoperatively [99]. Moreover, resection of the two pleural sheets causes the lung parenchyma to adhere to the thoracic wall, so that recurrent pleural effusion is rarely observed [100]. After completion of PD, a so-called hyperthermic intrathoracic chemoperfusion (HITOC) with cisplatin at 42 °C can be performed in the same surgery [89,101]. Hyperthermia results in sensitization of residual remaining tumor cells, and furthermore, a greater penetration depth of the chemotherapeutic agent is achieved and induction of signaling pathways for tumor cell apoptosis enabled [101]. Thus, PD in combination with HITOC opens up an adequate surgical treatment option for the increasing number of elderly patients with pleural mesothelioma, and does not appear to be inferior to the larger, radical procedure of EPP in terms of overall survival [93,98]. Compared with EPP, the perioperative morbidity of PD is lower, with the literature data ranging from 27.9% to 65%. In addition, it shows an acceptable 30-day mortality rate of 0% to 2.9% [93,102]. Due to the lack of a randomized trial, the choice of surgical intervention within the multimodal treatment scenario should be driven by comorbidities, clinical tumor stage, patient’s preference, and the experience of the respective center. As already described, the most important goal should always be MCR, as this indicates an important prognostic factor with regard to overall survival [103]. Another key feature is the patient’s quality of life during and after therapy. The existing literature shows an improvement in quality of life after P/D compared to EPP [99].

### 3.3. Surgical Methods in Palliation

According to the guidelines, there is clear consensus on the role of surgery in diagnosis, as mentioned above, and palliative therapy. The MesoVATS study, a randomized trial comparing talc pleurodesis with VATS partial pleurectomy (pp), reported no difference in one-year survival [104]. This has led to the consentaneous recommendation of prioritizing talcum over VATSpp [31,85,96]. However, VATSpp is preferred in patients fit for surgery with an entrapped lung. Whether this represents a benefit over the less complex intrapleural catheter in the entrapped lung is currently being investigated in a phase 3 trial (MesoTRAP) [105,106].

## 4. Systemic Treatment

### 4.1. Firstline Treatment

#### 4.1.1. Systemic Chemotherapy

Until very recently, cisplatin-based chemotherapy with pemetrexed represented the cornerstone in treatment of mesothelioma since it has been the sole approved treatment since 2004 [7]. There have been a number of studies investigating various chemotherapeutic or targeted drugs in different regimens since then. Unfortunately, a clinically meaningful improvement was still lacking [9,107,108,109,110]. A combination of cisplatin and pemetrexed with vitamin B12 and folic acid supplementation was the first chemotherapeutic doublet regimen to improve OS when compared with cisplatin alone (12.1 vs. 9.3 months; hazard ration [HR] 0.76). However, carboplatin is frequently used instead of cisplatin whenever the latter is contraindicated or is termed as more toxic. On the basis of multiple phase 2 studies and a meta-analysis that found no significant difference in OS or PFS, carboplatin and pemetrexed represent a good option with less non-hematological toxicity [111,112,113]. In another phase 2 trial, MAPS, a French research group showed improvement in OS and QoL with the addition of bevacizumab to cisplatin and pemetrexed when compared with cisplatin and pemetrexed alone (OS 18.8 vs. 16.1 months, HR 0.77). However, increased toxicities with the bevacizumab combination were reported: grade 3/4 hypertension (23% vs. 0%), grade 3 proteinuria (3.1% vs. 0%) and grade 3/4 thrombotic events (6% vs. 1%) [11]. Despite positive trial results, an approval was not sought either in Europe or the USA for this combination.

In contrary to non-small cell lung cancer (NSCLC) with non-squamous histology, where maintenance treatment with pemetrexed improves OS, there is no evidence for maintenance pemetrexed in pleural mesothelioma [114]. However, the Dutch trial NVALT19 did show a PFS benefit with gemcitabine switch maintenance after at least four cycles of platin-based chemotherapy with pemetrexed when compared with BSC (6.2 vs. 3.2 months, HR 0.48; *p* = 0.0002), without improving OS. Grade 3/4 adverse events (AE) were reported in 52% of the patients in the gemcitabine arm versus 16% in the supportive care arm [115]. In a merlin-stratified study, defactinib did not show any benefit (neither OS nor PFS) in maintenance treatment [116].

A phase 2 trial, TALAMESO, is evaluating maintenance treatment with talazoparib, a PARP inhibitor, given orally for two years following first-line chemotherapy among all three histological subtypes in pleural and peritoneal mesothelioma (NCT04462809).

#### 4.1.2. Tumor Treating Fields

Tumor Treating Fields (TTFields) have been studied in the treatment of pleural mesothelioma. TTFields use low intensity, alternating electric fields typically at a frequency of 150 kHz for their antimitotic effects and additionally thermic cytotoxic effect. Based on the data of the STELLAR trial, a phase 2 study that investigated its efficacy together with chemotherapy in first-line setting, TTFields were approved by the European medicine agency (EMA) and US Food and Drug Administration (FDA) under the humanitarian device exemption pathway. AE related to TTFields particularly was skin reaction, which occurred in 66% of patients as grade 1 and 2. Five percent of the patients experienced a grade 3 skin reaction [117]. However, lack of significant toxicity should not exempt a novel treatment strategy like TTFields from a confirmatory randomized phase 3 study. It should be noted that the reported OS (17.6 months), PFS (7.6 months) and RR (40%) are comparable with control arms receiving standard chemotherapy in recent randomized trials [11,118]. Similarly, high financial costs and impact on the quality of life due to continuous use of the device (up to 18 h daily) need to be taken into account.

#### 4.1.3. Immune Checkpoint Inhibition

Since the advent of ICI in solid tumors, including thoracic malignancies, multiple smaller trials with anti-PD1 or anti-PDL1 inhibitors have shown some activity in the salvage treatment pleural mesothelioma. Most of them primarily showed promising PFS, OS and disease control rate (DCR) as single agents [12,13,14,15,16,17,18]. Interestingly, tremelimumab, an anti-CTLA4-inhibitor, alone failed to show any benefit over placebo in previously treated patients [18]. However, positive results were reported with the combination of nivolumab and ipilimumab in a non-comparative trial (MAPS-2) in the context of salvage treatment after the failure of first-line treatment (RR 28%, DCR 52%; mPFS of 5.6 months) [13,119].

The randomized phase 3 trial, Checkmate-743, compared the combination of nivolumab and ipilimumab for up to two years or until progression or unacceptable toxicity with cisplatin or carboplatin and pemetrexed for up to six cycles in a first-line setting (Table 1). This study enrolled 605 treatment-naïve patients (eastern cooperative oncology group (ECOG) ≤1, epithelioid subtype ~75%). The OS was statistically improved to 18.1 months for the ICI combination, compared with 14.1 months for chemotherapy (HR 0.74). The OS did not differ significantly with ICI combination between patients with epithelioid subtype (18.7 months) and non-epithelioid subtype (18.1 months). When compared with chemotherapy, the most substantial survival advantage in favor of ICI revealed in the non-epithelioid subtype (8.8 vs. 18.1 months; HR = 0.46—epithelioid subtype 16.5 vs. 18.7 months; HR = 0.86). The positive expression of PD-L1 (≥1%) resulted in a meaningful improvement in OS of 18.0 vs. 13.3 months (HR = 0.69). However, a numerical increment in OS was also reported for PD-L1 <1% (17.3 vs. 16.5 months, HR = 0.94). Grade 3 or 4 AE were more common with ICI combination than with chemotherapy (15% vs. 6%, respectively) [20]. Treatment discontinuation owing to AE was higher with ICI combination than with chemotherapy (23% vs. 16%, respectively). Based on these data, the combination of nivolumab and ipilimumab was approved by FDA and EMA for the first-line treatment of non-resectable patients with pleural mesothelioma. Recently, a 3-year update was presented confirming the persisting benefit of the ICI combination over the chemotherapy doublet. The 3-year OS rate stood at 23% with ICI combination vs. 15% with chemotherapy. No significant difference in median PFS was reported between ICI and chemotherapy (6.8 versus 7.2 months, respectively; HR 1.00). However, at least 28% of the patients who received the ICI combination showed persisting response at three years, compared with 0% of the patients in the chemotherapy arm, despite no benefit in median ORR between the arms. TMB did not correlate with the survival benefit [120]. It must be noted that for the epithelioid subtype, platin/pemetrexed chemotherapy still represents a reasonable treatment option in the first-line setting. It is sensible to compare duration of treatment, toxicities and financial burden when ICI combination or chemotherapy is discussed in patients with epithelioid subtypes. Furthermore, the 3-year updated data also gave room for new questions: do patients benefit from an ICI and chemotherapy combination rather than ICI only, since PFS during the first 7 months of treatment with ICI was inferior to chemotherapy only? Can a subgroup be selected and tailored to receive best treatment approach based on novel biomarker?

First promising data have been presented for chemotherapy in combination with single agent ICI from single arm phase 2 studies. The DREAM trial was first to report a positive PFS result with cisplatin, pemetrexed in combination with durvalumab (mPFS 6.2 months). Grade 3 or 4 AE were observed in 66% of the patients. Fifteen percent reported immune-related grade 3 or 4 AE [121]. The same combination (carboplatin was allowed) demonstrated a promising OS (20.6 months) result in another single arm phase 2 trial [122]. The ongoing randomized DREAM3R trial (NCT04334759) (Table 1) is investigating the efficacy of chemotherapy and durvalumab in first-line treatment. As the prespecified secondary endpoint, this study also aims to investigate health care cost with regard to hospitalization, scheduled visits and medication. The results will help explore cost-effectiveness of such treatment. Another major randomized phase 3 trial (NCT02784171) is comparing cisplatin/pemetrexed and pembrolizumab with cisplatin/pemetrexed alone.

After seeing exciting results from chemoimmunotherapy and chemo-antiangiogenic strategies in the front-line setting, there is a logical rationale to investigate the combination of chemotherapy, checkpoint inhibitor and anti-angiogenic inhibitor. Such a regimen is already approved in the NSCLC. The phase 3 trial, BEAT-Meso (Table 1), aims to randomize 320 patients in first-line treatment who receive platin, pemetrexed and bevacizumab with or without atezolizumab. The primary co-endpoints are OS and PFS. Considering historical data, it is justified to assume that this combination (with or without Atezolizumab) may add a treatment option for at least, patients with epithelioid subtype. 

### 4.2. Salvage Treatment

#### 4.2.1. Chemotherapy Regimens

BSC, re-challenge of platin/pemetrexed or pemetrexed monotherapy have been the major domain of salvage treatment in mesothelioma amid a scarcity of treatment options [123,124,125,126]. However, based on retrospective data, single agent chemotherapeutics, vinorelbine or gemcitabine, have also been used as off-label drugs [8,127,128]. These prevalent chemotherapeutic options were supported and extended by recent phase 2 trials. The Italian RAMES trial compared gemcitabine with or without ramucirumab in patients who had progressed during or after first-line platin and pemetrexed. The combination treatment resulted in a significant improvement in OS (13.8 vs. 7.5 months, HR 0.71) and PFS (6.2 vs. 3.3 months) [129]. The VIM trial, for the first time, showed PFS benefit of vinorelbine plus active symptom control (ASC) over ASC only. The PFS was 4.2 months for patients who received vinorelbine and ASC. By contrast, patients with ASC only achieved PFS of 2.8 months, resulting in 40% risk reduction in disease progression or death with vinorelbine and ASC (HR 0.59). However, OS was not improved (9.3 vs. 9.1 months; HR 0.79 [95% CI 0.53–1.17]). In preclinical models, *BRCA1* was shown to regulate spindle checkpoint and a sensitivity to vinorelbine was predicted. However, in VIM trial, no association between *BRCA1* status and vinorelbine efficacy was found [130].

#### 4.2.2. Immune Checkpoint Inhibition

As mentioned earlier, small cohort studies initially provided some signal that single agent ICI may have some role in salvage treatment. The PROMISE-Meso trial (phase 3) recruited 144 patients who had progressed on or after first-line treatment and randomized 1:1 to receive either pembrolizumab or chemotherapy. The latter included gemcitabine or vinorelbine according to the physician’s choice. The primary endpoint of this trial was PFS by independent central review, which was not achieved with pembrolizumab (3.4 months for chemotherapy vs. 2.5 months for pembrolizumab, HR 1.06). The duration of response also supported chemotherapy (11.2 vs. 4.6 months). However, the objective response rate (ORR) was markedly improved with pembrolizumab than with chemotherapy (22% vs. 6%). It should be noted that 63% of the patients who progressed under chemotherapy did crossover to receive pembrolizumab. Nevertheless, no difference in the OS was observed (HR 1.04) even after adjusting for crossover [131].

Based on the results of a phase 2 study, nivolumab was approved as a second-line treatment in Japan in 2019 [132,133]. The CONFIRM trial compared nivolumab with placebo in patients who had experienced disease progression after first-line treatment. In this trial, 332 patients were randomly assigned (2:1) to receive either nivolumab or placebo. The co-primary endpoints of this study were PFS and OS. The majority of the patients had an epithelioid subtype (88%) and a negative PD-L1 tumor proportion score (TPS; <1%) (66%). More than half of the patients received nivolumab as their third-line treatment. The trial was positive in improving both PFS (3 vs. 1.8 months) and OS (9.2 vs. 6.6 months, HR 0.72; *p* = 0.018). The 1-year survival rate was reported to be 39.5% with nivolumab and 26.9% with the placebo. Surprisingly, 42% of the patients who received the placebo reported to have experienced grade 3 or 4 AE compared to 45% with nivolumab [134]. The double-blind study design probably contributed in such a tendency to attribute a higher number of AE in the placebo arm, underlining the impact of blinded study on the proper comparison of treatment arms. Since patients with prior treatment with an ICI were excluded from this study, the exact clinical efficacy of nivolumab is unclear in patients who have already received ICI. This represents a challenge in decision-making since the approval of nivolumab and ipilimumab in first-line setting, as the above-mentioned salvage trials were conducted in patients who were refractory to chemotherapy only. There seems to be a subgroup of patients with MPM who derive benefit from ICI, but a predictive biomarker is still missing. Due to the recent approval and ongoing promising trials in first-line setting (e.g., DREAM3R, BEAT-Meso and Canadian Cancer Trial Group) that may further enrich options in frontline treatment, there is an urgent unmet need for novel trials that would help tailor salvage treatment based on predictive biomarkers.

Tremelimumab has been compared with placebo in a phase 2 trial (DETERMINE), despite missing the primary endpoint (ORR) in a small single arm trial previously. The rationale for multi-national DETERMINE trial was based on the clinical activity and non-significant toxicities observed in the prior study [135,136]. The enrolled patients had disease progression after one or two systemic treatments. Altogether, 571 patients were randomized and assigned (2:1) to either tremelimumab or placebo. The primary endpoint, OS, did not show any difference between the arms (7.7 vs. 7.3 months; HR 0.92, *p* = 0.41). The safety profile was consistent with historical data available for anti-CTLA4-inhibitors [18]. 

While the anti-CTLA4-inhibitor failed to show any benefit, multiple non-comparative phase 2 trials have demonstrated the clinical activity of combining it with an anti-PD1 or anti-PDL1-antibody in later lines of treatment. MAPS-2 and INITIATE trials looked at the combination of nivolumab and ipilimumab, while the NIBIT-Meso-1 trial (Table 2) explored durvalumab and tremelimumab. RR were marginally lower than 30%, while DCR spread between 52% and 68% [13,14,119]. Recently, 4-year update data showed survival rates of 53% at 12 months, 35% at 18 months, 20% at 36 months and 15% at 48 months. At 52 months’ median follow-up, the median OS was 16.5 months [137,138].

### 4.3. Systemic Treatment in Resectable Stage

Basically, multimodal treatment in mesothelioma includes radical surgery with extended pleurectomy and decortication with the aim of achieving MCR, chemotherapy (platin and pemetrexed) with or without radiotherapy. Whether neo-adjuvant or adjuvant chemotherapy approach is better is a matter of current investigation. The EORTC trial is trying to address this question through an ongoing randomized clinical trial [139]. Historically, radiotherapy to the hemithorax used to be offered alongside EPP and chemotherapy [30,140,141,142]. However, since the radical surgery has left EPP and moved towards lung-preserving ePD, radiotherapy represents a risk for pneumonitis [30,143]. A less toxic novel method of radiotherapy, intensity modulated radiotherapy (IMRT), is currently being explored in a phase 3 trial [144,145].

#### Immune Checkpoint Inhibitors

The ongoing phase 2 NICITA trial investigates the feasibility and efficacy of nivolumab and chemotherapy as an adjuvant treatment, and is currently recruiting patients (*n* = 92) who have already undergone surgery by means of ePD. The patients are stratified according to ECOG performance status, MCR status and HITOC status, and then randomly assigned 1:1 to receive four cycles of platin and pemetrexed with or without nivolumab. After four cycles of chemoimmunotherapy in the interventional arm, nivolumab is continued four-weekly as a maintenance treatment for an additional one year [146]. Another trial, AtezoMeso, is evaluating atezolizumab as a maintenance adjuvant treatment. 

Other smaller trials are exploring neo-adjuvant ICI as a single agent (pembrolizumab, nivolumab) in combination with chemotherapy (atezolizumab) or another ICI (nivolumab and ipilimumab, durvalumab and tremelimumab) (Table 3). The result of these trials, together with those from the MARS-2 trial, will elucidate the role of multimodal treatment with ICI and/or chemotherapy.

### 4.4. Further Trials of Interest and Future Perspectives

A randomized phase 2–3 study of dendritic cells (DC) loaded with allogenic tumor cell lysate is being investigated in the DENIM trial (NCT03610360). After chemotherapy, patients are randomly assigned to receive the DC as a maintenance therapy alongside BSC or BSC only. The primary endpoint of this study is OS [23,147].

Studies targeting mesothelin, a cell surface marker predominantly found in mesothelioma, have also been conducted. These include an anti-mesothelin antibody with or without a drug conjugate and CAR-T cells. The latter has been explored in a phase 1 trial in combination with pembrolizumab, achieving DCR of almost 60% [26,27,148,149].

The INFINITE Study (NCT03710876), phase 3, is investigating the efficacy and safety of intrapleural administration of adenovirus-delivered interferon alfa-2b (rAd-IFN) in combination with oral celecoxib and gemcitabine on the basis of a prior phase 2 study, which had shown an RR of 25% and a DCR of 87.5%. The control group received oral celecoxib and gemcitabine only. The patients must have received at least one prior systemic treatment. The primary endpoint of this study is OS. However, this approach may not be feasible for patients without the possibility of placing an intrapleural catheter.

Better understanding of the genomic landscape, mutations and prevailing biology has revealed possibilities for targeted therapies in mesothelioma [33,48,150,151,152,153,154,155]. The MiST trial is evaluating treatments tailored on the basis of molecular stratification. The multiple arm assignment includes treatment with PARP inhibitor for *BRCA1*/*BAP1* negative disease, CDK4/6 inhibitor for p16ink4A negative, PD1 inhibitor in combination with AXL inhibitor without any specific biomarker, PDL1 inhibitor with VEGF inhibitor for PDL1 expression positive, and IG antibody with PARP inhibitor for platin sensitive disease. The first arm of study (MiST1) with rucaparib in patients with *BAP1* or *BRCA1* deficiency was reported to have reached its primary endpoint (DCR or 58% and 23% at 12 weeks and 24 weeks, respectively). Rucaparib was well tolerated with 9% grade 3/4 toxicities. Further investigation with PARP inhibitor is warranted in mesothelioma with homologous recombination deficiency associated with *BRCA1* mutation [156]. In recently published MiST2 trial, 26 patients with p16ink4A-deficient mesothelioma were treated with abemaciclib. DCR at 12 weeks was reported in 14 (56%) of the patients and thus the primary endpoint was met. Grade 4 or worse AE was reported in 12% of patients, serious AE in 23% and one patient died from neutropenic sepsis [157]. Due to molecular stratification, this study will help ascertain valuable knowledge in the targeted treatment of mesothelioma.

In patients with loss of argininosuccinate synthetase 1 (ASS1), arginine deprivation has been shown to be a promising approach that can be achieved with pegylated arginine deiminase (ADI-PEG20). Loss of ASS1 is common in the non-epithelioid subtype. After positive clinical activity in a phase 2 trial, an ongoing phase 3 trial is comparing chemotherapy with or without ADI-PEG20 (NCT02709512) in 386 patients [158,159]. Tazemetostat, an enhancer of the Zeste-Homolog2 (EZH2) inhibitor in *BAP1* inactivated mesothelioma, represents a further attempt in targeted therapy [116]. In Table 4. some ongoing trials are listed.

Mesothelin, a transmembrane tumor antigen, is highly expressed in solid cancers including mesothelioma, pancreatic and ovarian cancers [160]. It has been investigated as a possible therapeutic target in mesothelioma, including CAR-T therapy and antibody-drug conjugate (ADC) [161,162,163]. Anetumab ravtansine, an anti-mesothelin-specific ADC, was reported to show encouraging antitumor activity with manageable safety profile in pre-treated patients with mesothelioma in a phase 1 study [164]. Currently, a phase 1/2 study is comparing pembrolizumab with or without anetumab ravtansine in mesothelin-positive pleural mesothelioma (NCT03126630).

## 5. Conclusions

There are challenges in investigating pleural mesothelioma that are not limited to low incidence, individual heterogeneity of disease, lack of well-designed randomized trials, and non-standardized treatment approach, especially in the resectable stage. In meticulously selected patients with resectable disease, multimodal treatment should be approached in high volume centers, preferably in a clinical trial.

With an increasing number of effective drugs and treatment strategies, there have been some significant developments in recent years. The combination of nivolumab and ipilimumab outperforms platin and pemetrexed in terms of both overall survival and quality of life. The latter, however, continues to offer a robust treatment option especially for patients with the epithelioid subtype. The clinical benefit of nivolumab and ipilimumab is markedly impressive in the non-epithelioid subtype. The correct identification of the histological subtype is thus indispensable for treatment decision-making. Recent approvals and novel options in systemic treatment have been filling up the therapy arsenal with clinically meaningful results (Checkmate-743, RAMES, VIM, CONFIRM). However, there is an unmet need for effective treatment in refractory disease, which must be prioritized in future research.

Mesothelioma, as we have learnt, is not a disease prone to being tackled by the immune checkpoint inhibitor alone. Various trials with exciting combination approaches, including chemoimmunotherapy, combination of anti-VEGF inhibitors, tyrosine kinase inhibitors, and novel strategies incorporating CAR-T cells, oncolytic viruses and immunotoxins, are ongoing. The understanding of the tumor microenvironment and its impact on tumor proliferation, drug resistance and interplay with immune cells may reveal new possibilities in tailoring the future therapeutic landscape. The TME interaction in mesothelioma is a field of interest, and could act as a model disease to study the prerequisites of identifying subgroups that benefit from ICI, targeted/combination therapies and other TME-directed strategies. Better understanding of molecular profile, tumor-microenvironment, identification of predictive biomarkers and stratified treatment approach may help corroborate new treatment strategies, improve survival rate and quality of life of patients, and certainly ensure better research in the future.

## Figures and Tables

**Table 1:** Selected phase 3 randomized clinical trials in front-line treatment.

Trial	Drug	n	Maintenance	Histo	PE	PRO/QoL
NCT02899299 (Checkmate-743)	Nivolumab + Ipilimumab vs. Cisplatin or Carboplatin + Pemetrexed	605	Nivolumab + Ipilimumab for up to of 2 years *	All	OS	EQ-5D-3L, LCSS-Meso
NCT02784171 (Canadian Cancer Trials Group)	Pembrolizumab + Cisplatin + Pemetrexed vs. Cisplatin + Pemetrexed	520	Pembrolizumab for up to 2 years *	All	OS	QLQ-C30
NCT04334759 (DREAM3R)	Durvalumab + Cisplatin or Carboplatin + Pemetrexed vs. Cisplatin or Carboplatin + Pemetrexed	480	Durvalumab for up to 12 months *	All	OS	EQ-5D-5L, QLQ-LC29
NCT03762018 (BEAT-Meso)	Bevacizumab + Atezolizumab + Carboplatin + Pemetrexed vs. Carboplatin + Pemetrexed + Bevacizumab	320	Bevacizumab + Atezolizumab *	All	OS	PRO/QoL

NCT = ClinicalTrials.gov identifier; n = number of enrolled patients; Histo = histological subtype; PRO = patient reported outcome; QoL = quality of life; OS = overall survival; PFS = progression free survival; LCSS-Meso = Lung Cancer Symptom Scale (LCSS)-Mesothelioma; EQ-5D-3L = European Quality of Life 5 Dimensions 3 Level Version; ED-5D-5L = European Quality of Life 5 Dimensions 5 Level Version; QLQ-C30 = EORTC Quality of Life Lung Cancer Module-C30; QLQ-LC29 = EORTC Quality of Life Lung Cancer Module-LC29; * = Until progressive disease or unacceptable toxicities.

**Table 2:** Selected phase 2 trials with immunotherapy combinations in salvage treatment.

Trial	MAPS-2	INITIATE	NIBIT-Meso-1
Drug	Nivolumab + Ipilimumab	Nivolumab + Ipilimumab	Durvalumab + Tremelimumab
n	62	34	40
PR (%)	28	29	28
DCR (%)	52	68	65
PD (%)	42	32	35
mPFS (mo.)	5.6	6.2	8
mOS (mo.)	15.9	NR (12.7-NR)	16.6

n = number of enrolled patients; PR = partial remission; DCR = disease control rate; PD = progressive disease; mPFS = median progression free survival; mo. = months; mOS = median overall survival; NR = not reached.

**Table 3:** Selected trials with multimodal treatment and immune checkpoint inhibitors at an early stage.

Trial	*n (-N)*	Neoadjuvant	Adjuvant
NCT04177953 (NICITA)	92 1:1	none	Platin/Pemetrexed ± Nivolumab
NCT04996017	162 2:1	Chemotherapy allowed	Chemotherapy allowed + Atezolizumab
NCT02707666	15	Pembrolizumab	Platin/Pemetrexed
NCT03228537	28	Atezolizumab + Platin/Pemetrexed	Radiotherapy
NCT02592551	20		none
	−8	Durvalumab,	
	−8	Durvalumab + Tremelimumab,	
	−4	Placebo	
NCT03918252	30		none
	−15	Nivolumab	
	−15	Nivolumab + Ipilimumab	

NCT = ClinicalTrials.gov identifier; n = number of total patients to be enrolled; N = number of patients in treatment arms.

**Table 4:** Selected ongoing trials in salvage treatment.

NCT Number	Trial	Abbreviation	*n*
NCT04287829	Pembrolizumab plus lenvatinib in second line and third line malignant pleural mesothelioma patients	PEMMELA	36
NCT05005429	Study of the efficacy and safety of the bintrafusp alfa in previously treated advanced malignant pleural mesothelioma	BIMES	47
NCT03126630	Pembrolizumab with or without anetumab ravtansine in treating patients with mesothelin-positive pleural mesothelioma		110
NCT02709512	Phase 2/3 study in subjects with MPM to assess ADI-PEG 20 with pemetrexed and cisplatin	ATOMIC	386
NCT04300244	Nivolumab and ipilimumab +/− UV1 vaccination as second line treatment in patients with malignant mesothelioma	NIPU	118
NCT03710876	Efficacy & safety of rAd-IFN administered with celecoxib & gemcitabine in patients with malignant pleural mesothelioma	INFINITE	53

NCT = ClinicalTrials.gov identifier; n = number of total patients to be enrolled; ADI-PEG20 = pegylated arginine deiminase; rAd-IFN = adenovirus-delivered interferon alfa-2b.
